# Hyperglycemia Revisited: Deciphering Early Signaling Responses with ER-Stress-Related Effects and Connexins in the Spotlight

**DOI:** 10.3390/cells15181651

**Published:** 2026-09-13

**Authors:** Irgita Semini, Panagiotis Mihos, Aristi Volioti, Anastasia Rapti, Catherine Gaitanaki, Ioanna-Katerina Aggeli

**Affiliations:** Section of Animal and Human Physiology, Faculty of Biology, School of Science, National and Kapodistrian University of Athens, University Campus, Ilissia, 157 84 Athens, Greece; irgitasem03@gmail.com (I.S.); pnh.mihos@gmail.com (P.M.); aristivoliotis@gmail.com (A.V.); anastasia1rapti@gmail.com (A.R.); cgaitan@biol.uoa.gr (C.G.)

**Keywords:** hyperglycemia, cardiac cells, apoptosis, oxidative stress, autophagy, ER stress, connexin 43, GJA1-20k, MAPKs

## Abstract

**Highlights:**

**What are the main findings?**

- The present study sheds light on the early high-glucose-activated signaling pathways in H9c2 cardiac cells related to ERK1/2, p38-MAPK, heme oxygenase-1, autophagy, ER stress, connexin 43 and GJA1-20k.
- Our findings indicate the detrimental input of apoptosis and oxidative stress in addition to the salutary effect of ER-stress-associated mediators and ERK1/2 activation in H9c2 cells exposed to high glucose levels.

**What are the implications of the main findings?**

- Gradual induction of connexin 43 and GJA1-20k by hyperglycemic conditions has not been shown before in cardiac cells, indicating the potentially fundamental role of these biomechanical sensors in the hyperglycemic milieu.
- The modulatory impact of ER-stress-related effectors and ERK1/2 on high-glucose-induced connexin expression levels may provide candidate targets for novel therapeutic interventions against adverse hyperglycemia-associated cardiovascular complications.

**Abstract:**

Diabetes constitutes one of the major prevailing diseases worldwide, with cardiovascular pathologies as the primary cause of the morbidity and mortality rates reported. Hyperglycemia, the principal hallmark of diabetes, results from accumulated glucose levels. Although molecular mechanisms induced by high glucose (HG) have been extensively investigated, their complex interconnections and immediately activated signaling pathways remain unresolved. Hence, in the present study, we tried to identify effectors directly responsive to HG, focusing on the early activated signal transduction routes in H9c2 cardiac cells. MTT analysis illustrated the apoptosis- and oxidative-stress-mediated detrimental effect of 25 mM glucose on H9c2 viability. Initiation of oxidative-stress-related mechanisms was corroborated via detection of POR and HOX-1 augmented expression levels. Additionally, western blot analysis demonstrated activation of p38-MAPK and ERK1/2, along with autophagy- and ER-stress-related markers. Of note, involvement of biomechanical signaling players was also revealed, with Piezo1, connexin 43 and GJA1-20k expression being gradually enhanced. Intriguingly, ERK1/2 and ER-stress-associated effectors were observed to mediate connexin 43 and GJA1-20k upregulation. With connexins playing a nodal biological role in diabetes-driven cardiovascular pathologies, exploring potential modulatory effectors may provide insight into development of promising therapeutic interventions, favoring preservation of cell function and systems homeostasis under hyperglycemic conditions.

## 1. Introduction

Diabetes mellitus (DM) is a metabolic disorder defined by persistent hyperglycemia. According to the International Diabetes Federation, approximately 589 million people were diagnosed with diabetes in 2024 worldwide, with the number predicted to reach ~853 million by 2050. Type 1 diabetes (T1DM) accounts for ~10% of all cases, with the rest corresponding to T2DM (World Health Organization data). Complications, whether acute or chronic, include ketoacidosis, hyperosmolar hyperglycemic state, microvascular or macrovascular dysfunction promoting diabetic nephropathy and neuropathy, or cardiovascular disorders (CVDs) and arterial diseases, respectively [1]. Of note, CVDs are the leading cause of death among DM patients [2]. Despite the adoption of healthier lifestyles via engagement in appropriate exercise and diet routines and effective therapeutic strategies, understanding the underlying signaling mechanisms remains of fundamental significance in the quest for DM remission. To date, research has almost exclusively focused on chemically induced (with alloxan or streptozotocin) DM animal models or in vitro cell cultures persistently exposed to HG for more than 24 h [3,4]. Hence, these experimental approaches aim to explore the ultimate, long-term effects of hyperglycemia, with the signaling responses triggered early on remaining elusive.

Accumulating evidence has correlated hyperglycemia with pro-death processes propagating its detrimental impact, leading to development of diabetic cardiomyopathy or heart failure [5]. Cardiotoxicity conferred by hyperglycemia has been associated with excessive generation of reactive oxygen species (ROS), induction of autophagy and apoptosis [6]. When the oxidative equilibrium is disturbed, expression of heme oxygenase-1 (HOX-1) is boosted, as an adaptive defense system protecting tissues against the oxidative insult [7,8]. Protection conferred by HOX-1 is attributed to heme cleavage and subsequent formation of biliverdin, iron and CO [9]. HOX-1 is anchored to the endoplasmic reticulum (ER) plasma membrane, with the P450 oxidoreductase (POR), its essential electron donor, being located nearby.

Meanwhile, while autophagy and apoptosis are routes of opposing endings, they are closely interconnected and can share common triggering stimuli, including hyperglycemia [10], particularly in heart pathophysiological disorders [11]. Autophagy usually preserves cell survival via degradation and recycling of dysfunctional components, with AMPK serving as a cornerstone effector by directly phosphorylating ULK-1 [12]. Alternatively, apoptosis is a thoroughly investigated form of programmed cell death, executed either via the extrinsic (death-receptor-mediated) or the intrinsic (cytochrome c- and caspase 3-mediated) pathway, orchestrating elimination vs. proliferation [13].

In abrupt or severe metabolic shifts, these mechanisms are also intricately linked to ER stress, whose initiation coincides with accumulation of unfolded or misfolded proteins in the ER [14]. BiP (binding immunoglobulin protein) serves as a key ER chaperone, primary sensor of ER stress and trigger of the unfolded protein response (UPR) [15], leading either to restoration of balance or to detrimental pathologies [16]. ER stress, among other intrinsic or external insults, can also lead to activation of the integrated stress response (ISR) so as to encounter and withstand it [17]. Phosphorylation of eIF2 alpha is a pivotal marker of this adaptive signal-transducing response initiated to preserve cell homeostasis via halting protein translation, also found to coincide with ER stress under hyperglycemic conditions, so as to alleviate the discomfort [18]. ER stress plays a nodal role as a “surveillance system” that monitors any distortion of proteins’ proper folding, organelle functionality and calcium or redox equilibrium. It also coordinates lipid and glucose homeostasis. In their recent review, Wei et al. 2026 [19] focused on the plethora of disorders linked to ER stress, including cardiovascular diseases, neurodegenerative syndromes, metabolic perturbation, autoimmunity and cancer. Hence, it is of fundamental significance to explore its potential contribution to hyperglycemic complications via identification of previously unidentified routes it may affect.

Hyperglycemia can be assumed to act not only as a biochemical but also as a mechanical stressor in the heart. Conversion of mechanical cues to biological messages is mediated by Piezo channels, integrins, connexins, Yes-associated protein (YAP)/transcriptional coactivator with PDZ-binding motif (TAZ), etc. [20]. Piezo1, in particular, is a mechanosensitive cation channel directly involved in converting mechanical cues into calcium- and ROS-dependent signals, contributing to maintenance of normal heart function [21,22]. Of interest, low-intensity pulsed ultrasound (LIPUS) was found to alleviate symptoms of hyperglycemia in T1DM mice via Piezo1 activation [23]. Connexins are transmembrane proteins that also sense and transmit biomechanical forces within the cardiovascular system [24]; six connexins oligomerize into connexons that may function either as hemichannels, permitting diffusion of small molecules, or, if docked to an opposite connexon, may form gap junctions to synchronize responses or exchange ions, small metabolites and peptides (i.e., ATP, Ca^2+^, and cAMP) [25]. Among the ~20 members of the connexin family, connexin 43 is the primary one in mammalian heart ventricles. Intriguingly, it is encoded by *Gja1*, a gene that, via internal translation initiation, generates at least six isoforms truncated at the N-terminus and named after their predicted size. One of these isoforms, GJA1-20k (gap junction protein alpha 1 (20 kDa)), is the most abundantly expressed in the mammalian heart and contains part of the connexin 43 transmembrane domain plus its whole C-terminus tail [26]. Given that responses induced by hyperglycemia have been previously correlated with disrupted connexin functionality [27], assessing these responses is of utmost significance.

Collectively, in the present study, our goal was to decipher the transduction pathways that are activated by HG in H9c2 cardiac cells, placing emphasis on identifying, predominantly, the signaling effectors induced early on, considering their decisive contribution to the conclusive, adverse end-points of hyperglycemia, particularly in cardiac cells. To this end, H9c2 cardiac cells were exposed to high glucose levels, with the key difference concerning the duration of the stimulus, which, instead of the 24–72 h duration almost exclusively implemented previously, consisted of time intervals ranging from 5 min to 4 h. Activation of signaling pathways indicative of apoptosis, autophagy and ER stress was observed, along with the triggering of antioxidant mediators or mechanical-stress-related responses in order to counteract the hyperglycemic insult. *One of the core findings of the present study is the relatively rapid induction of connexin expression as well as of their subcellular redistribution by HG in an ER-stress-associated manner*. Our data are of nominal clinical value and further elucidate the potential crosstalk and interplay among all these pathways, essential so as to achieve enhancement of cell survival and secure cell viability under these conditions.

## 2. Materials and Methods

### 2.1. Reagents and Antibodies

AppliChem GmbH (Darmstadt, Germany) provided dithiothreitol (DTT), phenylmethylsulphonyl fluoride (PMSF), dimethyl sulfoxide (DMSO) and Bradford reagent. Leupeptin, trans-epoxysuccinyl-L-leucylamido-(4-guanidino) butane (E-64), melatonin, Ponceau S, acridine orange, U0126 and SB203580 were purchased from Merck (Burlington, MA, USA). Bafilomycin A1 and 4-phenylbutyric acid (4-PBA) were purchased from Sigma-Aldrich (St. Louis, MO, USA). ZVAD-FMK was provided by Cayman Chemical (Ann Arbor, MI, USA).

Nitrocellulose (0.45 μm) was bought from Macherey-Nagel GmbH (Duren, Germany). Prestained molecular mass markers were acquired from New England Biolabs (Beverly, MA, USA). The rabbit polyclonal antibodies for poly (ADP-ribose) polymerase (PARP) (#9542), cleaved PARP (#5625), caspase-3 (#9662), cleaved caspase-3 (#9661), BiP (#3183), eIF2α (#9722), phospho-AMPKα (Thr172) (#2535), AMPKα (#5831), phospho-eIF2α (Ser51) (#9721), phospho-p38-MAPK (Thr180/Tyr182) (#9211), p38-MAPK (#9212), phospho-p44/42 MAPK (Erk1/2) (Thr202/Tyr204) (#9101), p44/42 MAPK (Erk1/2) (#9102), heme oxygenase-1 (#43966), POR (#90426), SQSTM1/p62 (#5114) and phospho-Ulk-1 (Ser317) (#37762) were purchased from Cell Signaling Technology Inc. (Beverly, MA, USA), while rabbit polyclonal anti-actin (A2103) was purchased from Merck (Burlington, MA, USA). Piezo1 (#15939-1-AP) antibody was acquired from Proteintech (Planegg-Martinsried, Germany), and anti-connexin 43 antibody (C6219) was obtained from Merck. The peroxidase-conjugated goat anti-rabbit IgG secondary antibody (#AP132P) as well as the anti-rabbit IgG TRIC-conjugated secondary antibody (#T-5268) were also obtained from Merck. The enhanced chemiluminescence (ECL) kit was obtained from GE Healthcare (Buckinhamshire, UK). Super RX film was purchased from Fuji photo film GmbH (Dusseldorf, Germany). The cell culture supplies were obtained from PAA Laboratories (Pasching, Austria).

### 2.2. H9c2 Cell Culture and Treatment Regime

H9c2 rat cardiac myoblasts (passage 18−25; American Type Culture Collection CRL-1446, Manassas, VA, USA) were grown in Dulbecco’s modified Eagle’s medium (DMEM; Gibco, Paisley, UK) containing high glucose (4.5 g/L) in the presence of 10% (*v*/*v*) fetal bovine serum (FBS; ThermoFischer Scientific, Waltham, MA, USA) and penicillin–streptomycin (ThermoFischer Scientific, Waltham, MA, USA) under a humidified atmosphere of 95% air/5% CO_2_ at 37 °C. Cells were seeded in 60 mm dishes and grown to approximately 70% confluence in the aforementioned medium. Serum was withdrawn for at least 18 h prior to any treatment. With glucose already present (4.5 g/L ~ 25 mM) in the culture medium according to ATCC’s instructions to ensure euglycemia of the cells, exogenous supplementation of either 25 mM glucose or mannitol (as an osmotic control) was performed so as to investigate the effect of hyperglycemia in this particular cell line for the times indicated. All pharmacological inhibitors used were dissolved in DMSO and added to the medium 30 min prior to glucose: ZVAD-FMK (a known caspase inhibitor—20 μM), U0126 (an inhibitor of MEK1/2—15 μM), SB203580 (an inhibitor of p38-MAPK—10 μM), PBA (an established ER stress inhibitor—5 mM) and bafilomycin (an inhibitor of the late phase of autophagy—50 nM). Thus, H9c2 cells were left untreated (control), incubated with the inhibitors alone, or with the inhibitors followed by exposure to glucose for the times indicated. Alternatively, cells were treated with mannitol (25 mM) as an osmotic control so as to discriminate any osmotically induced responses from the ones attributed to glucose-elicited effects.

### 2.3. Cell Viability Assessment

The number of viable cells was evaluated by performing the 3-(4,5-dimethylthiazol-2-yl)-2,5-diphenyltetrazolium bromide (MTT) assay. Cells were seeded in 96-well plates (5 × 10^3^ cells/well) and were either left untreated (control) or treated with the inhibitors alone (as previously outlined) or with the inhibitors followed by treatment with glucose (25 mM). All experiments were performed in quadruplicate. After a 4 h incubation with MTT, the medium was aspirated, cells were lysed in 0.1 M HCl/isopropanol to dissolve the formazan crystals, and absorbance was measured at 545 nm in an ELISA microplate reader (DENLEY, West Sussex, UK) (*n* = 4).

### 2.4. Protein Isolation and Western Blot Analysis

After treatments, cells were washed twice with ice-cold phosphate-buffered saline (PBS) and harvested. For whole-cell samples, H9c2 cells were lysed in ice-cold buffer (20 mM Tris-HCl (pH 7.5), 20 mM β-glycerophosphate, 2 mM EDTA, 10 mM benzamidine, 20 mM NaF, 0.2 mM Na_3_VO_4_, 200 μM leupeptin, 10 μM E-64, 5 mM DTT, 300 μM PMSF and 0.5% (*v*/*v*) Triton X-100), followed by a 15 min incubation on ice. After centrifugation (BR4i Jouan centrifuge, 20.800× *g*, 10 min, 4 °C), the supernatants (total protein extract) were collected. The protein concentration was determined via the Bradford assay. After quantification, 0.33 vol. of sodium dodecyl sulfate (SDS) sample buffer (SB4X: 0.33 mol/L Tris-HCl (pH 6.8), 10% (*w*/*v*) SDS, 13% (*v*/*v*) glycerol, 20% (*v*/*v*) 2-mercaptoethanol and 0.2% (*w*/*v*) bromophenol blue) was added to the samples, which were boiled and stored at −20 °C. Samples for detection of caspase-3 activation were prepared with Chaps buffer (50 mM HEPES/KOH (pH 6.5), 2 mM EDTA, 0.1% (*w*/*v*) Chaps, 20 μg/mL leupeptin, 5 mM DDT, 1 mM PMSF, 10 μg/mL aprotinin and 10 μg/mL pepstatin A). After homogenization, the lysates were sequentially frozen (−80 °C (×3)) and left to thaw. Once again, after centrifugation of the lysates (20,800× *g*, 4 °C, 20 min), the protein concentrations were assessed using the Bradford assay. The supernatants were then boiled with 0.33 vol. of SB4X. The protein samples (40 μg of total protein) were run on SDS-PAGE on 8% (*w*/*v*), 10% (*w*/*v*) or 15% (*w*/*v*) polyacrylamide gels and transferred onto nitrocellulose membranes (0.45 μm). Ponceau staining was used in order to visualize transfer efficiency and equal protein loading. After blocking in TBST (Tris-buffered saline with Tween) containing 5% (*w*/*v*) non-fat milk powder (60 min; room temperature), the membranes were incubated overnight with the appropriate antibody (at a 1:1000 dilution). After incubating the membranes with the respective horseradish peroxidase-conjugated secondary antibody (1:5000 dilution), blots were developed using enhanced chemiluminescence (ECL) and quantified by scanning densitometry (Gel Analyzer v. 1.0). Normalization was carried out either by dividing the average value of each protein studied by the respective levels of the control protein (actin) in each sample, or via total protein normalization using Ponceau S staining [28].

### 2.5. Acridine Orange Staining

H9c2 cells were seeded on ibidi µ-Slide 4 Well coverslips (~80,000 cells/well, ibidi GmbH, Lochham, Germany). Before all treatments, the serum was withdrawn for at least 18 h. Subsequently, the H9c2 cells were left untreated (control) or treated with glucose (25 mM) for 1 and 2 h. The cells were then washed three times with PBS and incubated with acridine orange (5 μM in PBS) for 10 min at 37 °C in the dark. After washing the cells again three times with PBS, they were left in PBS and visualized under a Zeiss Axioplan microscope (Zeiss, Jena, Germany). Digitized images were acquired with a Zeiss Axiocam MRc5 digital camera with the AxiovisionRel 4.4 image software (Edition 2005).

### 2.6. Immunofluorescence Staining

Cells were grown on ibidi µ-Slide 4 Well coverslips (~80,000 cells/well). As previously stated, before all treatments, the serum was withdrawn for at least 18 h. Next, the H9c2 cells were left untreated (control), treated with high glucose (25 mM) or PBA (5 mM), or pre-treated with PBA for 30 min followed by exposure to glucose for 1 h in the presence of PBA (PBA/high glucose). After washing, the cells were fixed with 4% (*v*/*v*) formaldehyde in phosphate-buffered saline (PBS) at pH 7.4 (15 min; R_T_), washed in PBS (×3), and incubated (15 min; R_T_) with 1% (*w*/*v*) BSA in PBS containing 0.3% (*v*/*v*) Triton X-100. Incubation with the primary antibody against connexin 43 (1:100, O/N, 4 °C) was followed by washing with PBS and a 3 h incubation at 37 °C with a TRIC-conjugated anti-rabbit secondary antibody (1:40) (red fluorescence). After washing, the cells were visualized once again under a Zeiss Axioplan microscope. Digitized images were acquired with a Zeiss Axiocam MRc5 digital camera with the AxiovisionRel 4.4 image software (Edition 2005). Representative images are shown, indicative of at least three independent experiments.

### 2.7. Statistical Evaluations

Images of western blots presented were acquired from at least three independent experiments. In all cases, the data shown are presented as means ± SEM and were analyzed by a one-way ANOVA multiple-comparison test (Graph Pad Prism 8.4.3 Software, San Diego, CA, USA), with group comparisons performed using the Bonferroni post hoc test. *p* < 0.05 was considered to indicate a statistically significant difference.

## 3. Results

### 3.1. Hyperglycemia Exerts a Detrimental Effect on H9c2 Cellular Viability via Multiple Signaling Pathways and Mediators

**Figure 1:**
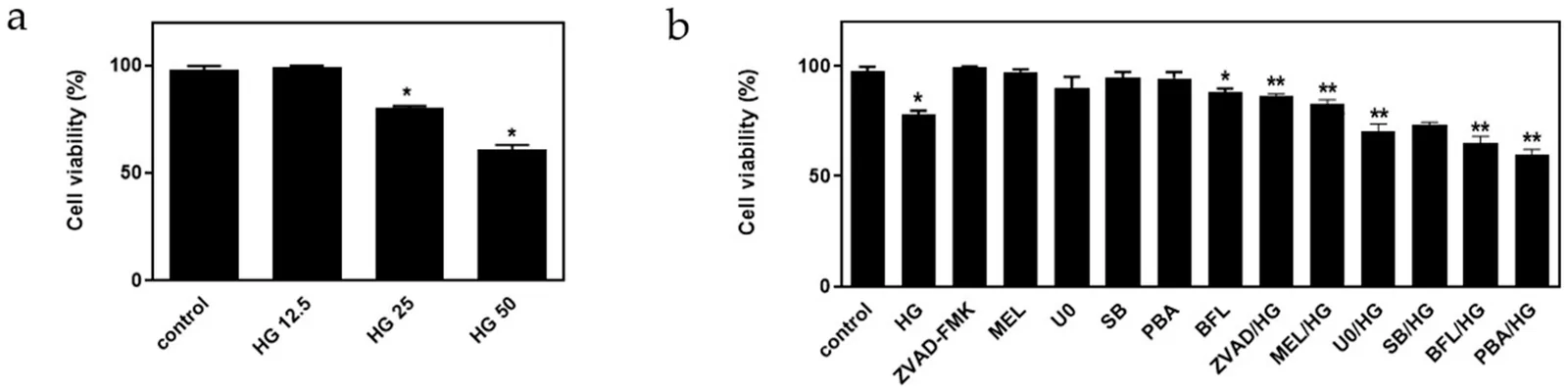
(**a**) Increasing concentrations of glucose gradually impair H9c2 viability. Measuring H9c2 (%) cell viability by MTT analysis, cells were left untreated (control) or were treated with exogenously added glucose at 12.5, 25 and 50 mM (HG) for 24 h. (**b**) ERK1/2 pathway, autophagy and ER stress appear to exert a beneficial effect during their induction in H9c2 cells treated with 25 mM glucose. For evaluation of H9c2 (%) cell viability by MTT analysis, cells were left untreated (control); treated with 25 mM glucose (HG) for 24 h; treated with ZVAD-FMK, melatonin, U0126, SB203580, bafilomycin and PBA alone for 24 h; or treated with ZVAD-FMK, melatonin, U0126, SB203580, bafilomycin and PBA for 30 min followed by exposure to 25 mM glucose (HG) for 24 h. For all experiments, *n* = 4. Values are presented as means ± SEM. * *p* < 0.05 compared with control values; ** *p* < 0.05 compared with HG-treated cells.

Given that high glucose (HG) levels are known to exert a deleterious effect on cell viability, we initially made an effort to assess this impact in our experimental setting. To this end, H9c2 cells were exposed to increasing concentrations of exogenously added glucose, exceeding the standard levels already present in the culture medium (control condition) by 12.5, 25 and 50 mM glucose for 24 h (Figure 1a; * *p* < 0.05 compared with control values). While 12.5 mM glucose had no apparent effect on cell viability, the latter was reduced by ~22 ± 0.65% in the presence of 25 mM glucose, with cell death reaching ~39 ± 1.37% in the presence of 50 mM glucose. Since a great number of studies have focused on the long-term effects of hyperglycemia, performing analyses in diabetic experimental animal models or studying responses in cells treated with HG for more than 24 h, the present study emphasized the early responses triggered, initiating signaling cascades that ultimately converge in the detrimental impact demonstrated in Figure 1a. Hence, we next sought to identify potential mechanisms involved. To this end, H9c2 viability was examined under hyperglycemic conditions in the presence or absence of ZVAD-FMK (a known caspase inhibitor—20 μM), melatonin (established for its antioxidative properties—10 μM), U0126 (an inhibitor of MEK1/2—15 μM), SB203580 (an inhibitor of p38-MAPK—10 μM), PBA (an established ER stress inhibitor—5 mM) and bafilomycin (an inhibitor of the late phase of autophagy—50 nM). Interestingly, compared with HG (78 ± 0.65%), cell viability was significantly enhanced in the presence of ZVAD-FMK (86.33 ± 0.98%) as well as melatonin (83 ± 0.78%), a result indicative of apoptosis and oxidative stress contribution to the adverse effect of hyperglycemic conditions. Conversely, the ERΚ1/2 pathway, along with autophagy- and ER-stress-associated mechanisms, appear to play a salutary role in H9c2 cells exposed to HG, since in the presence of their respective inhibitors, cellular viability was further repressed, reaching 70.3 ± 0.78%, 65 ± 0.77% and 59.6 ± 0.99%, respectively (Figure 1b; * *p* < 0.05 compared with control values, and ** *p* < 0.05 compared with HG-treated cells). Taking into consideration these data, we next sought to investigate in detail the time profile of established markers of the mechanisms found to be involved and the mediators identified to elicit the responses induced by HG herein.

### 3.2. High Glucose Triggers Apoptosis in H9c2 Cells via the Intrinsic Pathway

**Figure 2:**
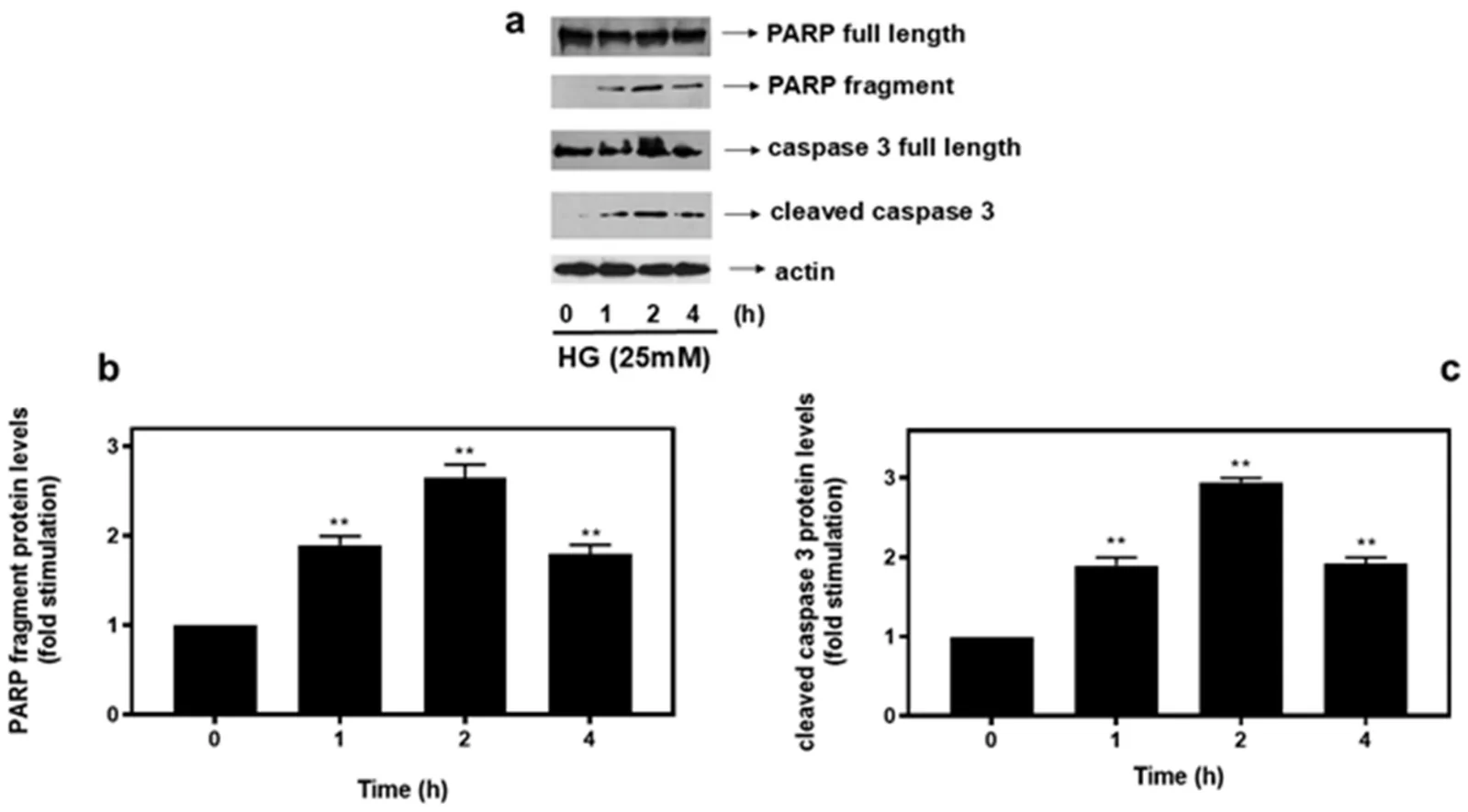
HG induces PARP proteolysis ((**a**) 2nd panel) and caspase 3 cleavage ((**a**) 4th panel from top) in a time-dependent manner in H9c2 cardiac cells. H9c2 cells were treated with 25 mM glucose (HG) for the times indicated. (**a**) Protein extracts (40 µg/lane) were subjected to SDS-PAGE and immunoblotted with antibodies for PARP (full-length and fragments: 1st and 2nd upper panels), for caspase 3 (uncleaved and fragment: 3rd and 4th panels from top), as well as for actin levels (bottom panel). Representative western blots of at least three independent experiments with overlapping results are illustrated. Quantification of protein bands was performed by scanning densitometry and plotted (respective graphs: (**b**) and (**c**)). Results are means ± SEM (n = 3); ** *p* < 0.01 compared with control values.

Corroborating the MTT analysis performed, many studies have already pinpointed apoptotic cell death as a mechanism that mediates hyperglycemic complications (reviewed in [29]). Accordingly, in western blot analysis we performed, proteolysis of PARP and of caspase-3 was observed as early as 1 h after exposure of cells to 25 mM glucose (Figure 2; ** *p* < 0.01 compared with control values), with maximal values attained at 2 h (2.65 ± 0.19- and 2.95 ± 0.1-fold relative to the control, respectively). As an internal control, an antibody against the housekeeping protein actin was used (Figure 2a, bottom panel).

### 3.3. High Glucose Upregulates Expression Levels of POR and HOX-1

**Figure 3:**
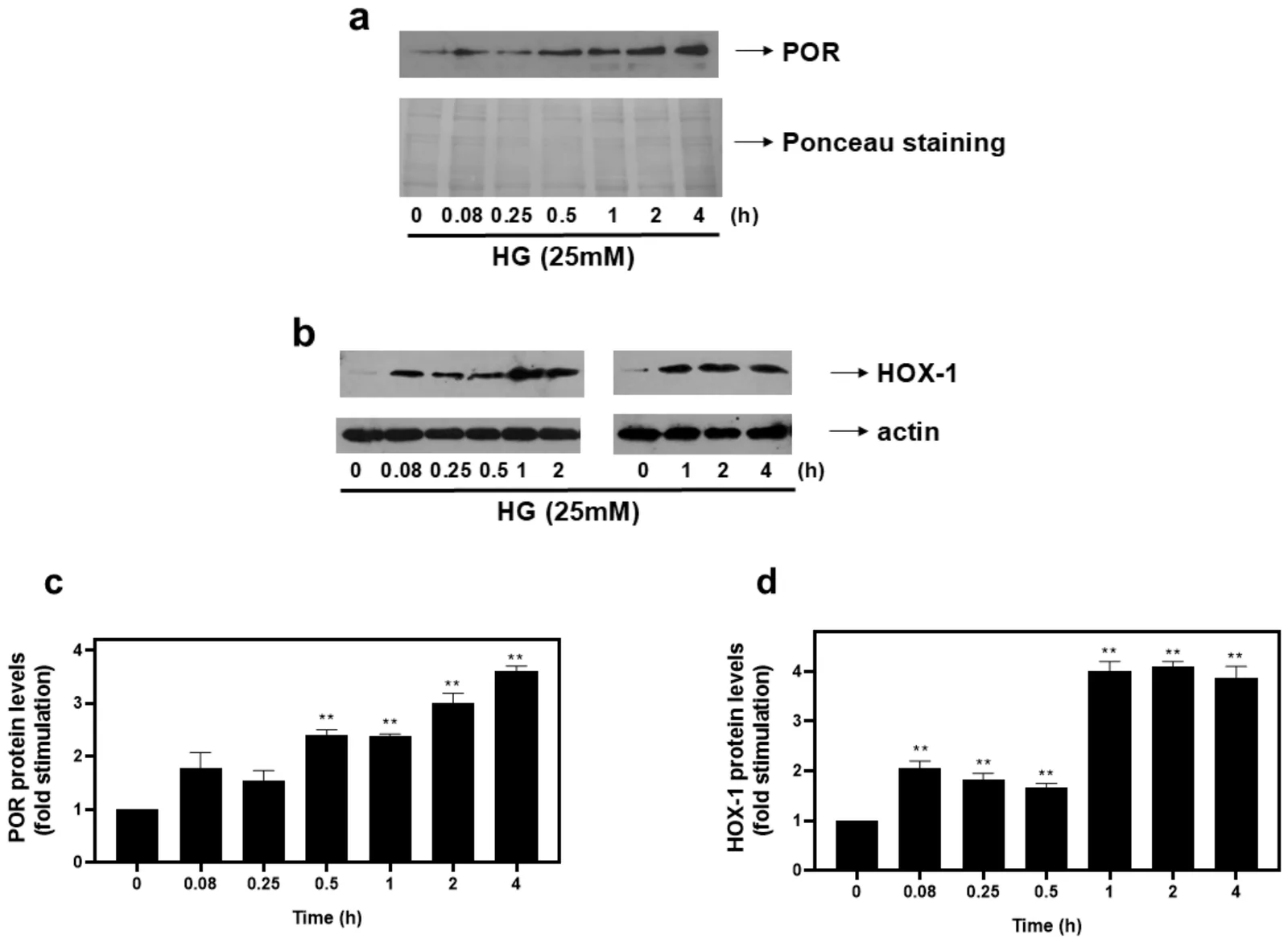
Time-dependent pattern of HG-induced POR (**a**) and HOX-1 (**b**) expression in H9c2 cardiac cells. H9c2 cells were treated with 25 mM glucose (HG) for the times indicated. (**a**) Protein extracts (40 µg/lane) were subjected to SDS-PAGE and immunoblotted with an anti-POR antibody (**upper panel**). Equal protein loading and transfer efficiency were verified via Ponceau staining of the respective membrane (**bottom panel**). (**b**) Protein extracts (40 µg/lane) were subjected to SDS-PAGE and immunoblotted with an anti-HOX-1 antibody (**upper panels**) and an anti-actin antibody (**bottom panels**). The actin blot presented in Figure 3b, left bottom panel, is part of the respective blot serving as an internal loading control, also illustrated in Figure 6, as they both correspond to identical sample lysates. The western blots shown are representative of at least three independent experiments with overlapping results. Quantification of protein bands was performed by scanning densitometry and plotted (respective graphs: (**c**) and (**d**)). Results are means ± SEM (n = 3); ** *p* < 0.01 compared with control values.

While cytochrome P450 oxidoreductase (POR) plays an established role as a donor of electrons for microsomal cytochrome P450 enzymes and other redox partners, including heme oxygenase-1 (HOX-1), interrelated responses of POR and HOX-1 are sparsely examined [30,31]. Given the identified input of oxidative stress in the responses induced by 25 mM glucose in our experimental setting (Figure 1b), we next set out to look into any potential effect of HG on both POR and HOX-1 expression levels in H9c2 cells. Interestingly, investigating the time course profile of POR, a gradual augmentation in POR expression was observed, with maximal values attained after 4 h of exposure to 25 mM glucose (3.6 ± 0.15-fold relative to the control; Figure 3a upper panel and Figure 3c; ** *p* < 0.01 compared with the control values). Meanwhile, HOX-1 expression levels were rapidly stimulated from as early as 5 min of treatment with 25 mM glucose and were maximized after 1 h, remaining elevated for at least 4 h (3.88 ± 0.19-fold relative to the control) (Figure 3b, upper panel, and Figure 3d; ** *p* < 0.01 compared with the control values). Equal protein loading was verified either by staining the membrane with Ponceau (Figure 3a, bottom panel) or by immunoblotting identical samples with a specific anti-actin antibody (Figure 3b, bottom panels).

### 3.4. High Glucose Induces Expression of Biomechanical Sensors in H9c2 Cells

**Figure 4:**
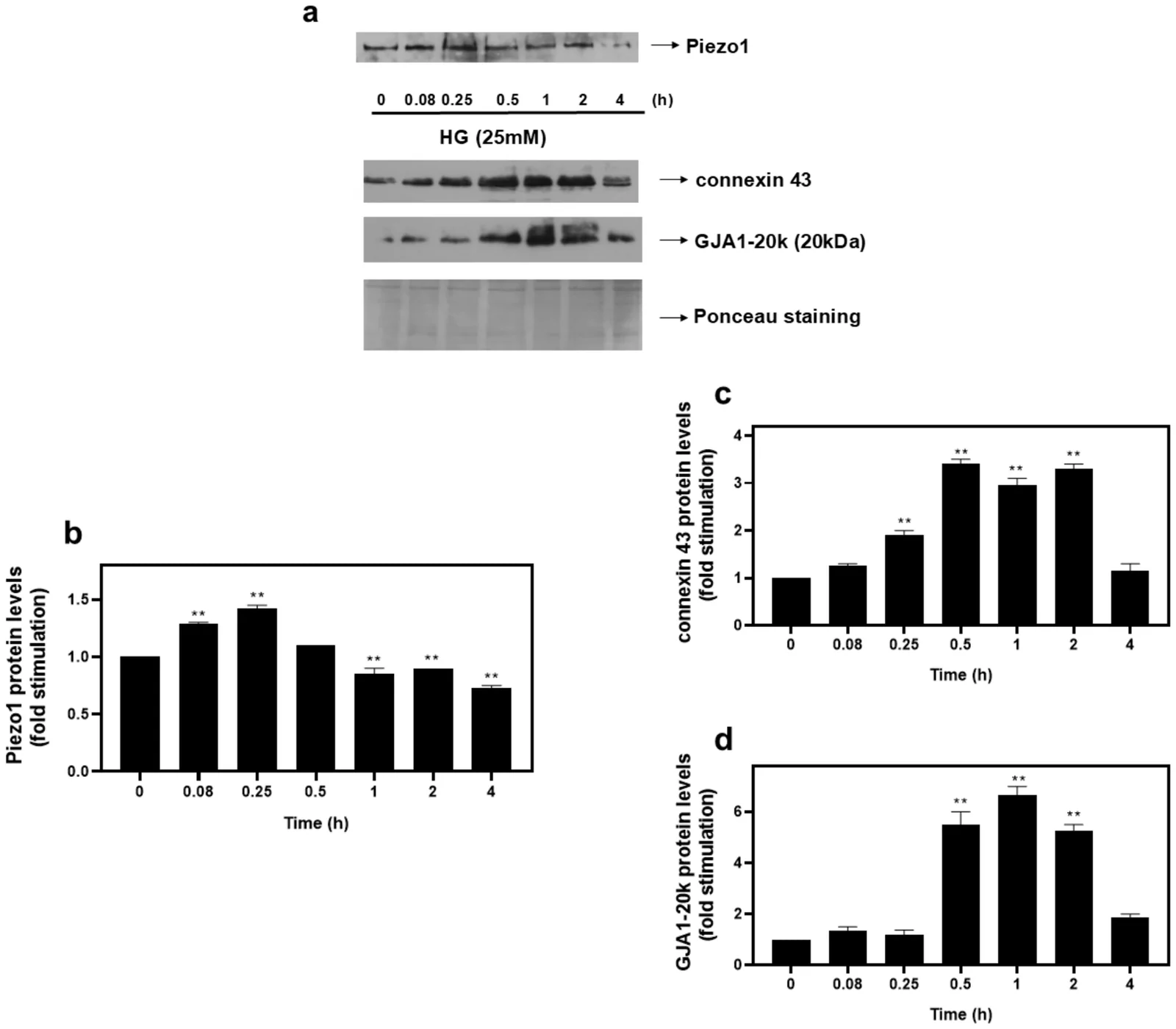
HG increases Piezo1 and connexins expression levels in a time-dependent manner. H9c2 cells were treated with 25 mM glucose (HG) for the times indicated. (**a**) Protein extracts (40 µg/lane) were subjected to SDS-PAGE and immunoblotted with an anti-Piezo1 antibody (**upper panel**). After SDS-PAGE, samples were also immunoblotted with an anti-connexin 43 antibody detecting connexin 43 (2nd panel from top) and GJA1-20k (3rd panel from top). To confirm equal protein loading and transfer efficiency, the respective membrane was stained with Ponceau (**bottom panel**). Representative western blots of at least three independent experiments with overlapping results are shown. Quantification of protein bands was performed by scanning densitometry and plotted (respective graphs: (**b**), (**c**) and (**d**)). Results are means ± SEM (*n* = 3). ** *p* < 0.01 compared with control values.

With high glucose observed to induce mechanotransduction-related responses in various cell types, i.e., endothelial cells or podocytes [32], in the long-term, we subsequently decided to investigate the effect of 25 mM glucose on nodal regulators of such processes in cardiac cells. In this context, we initially looked at the time-dependent expression profiles of Piezo1, a non-selective mechanosensitive ion channel known to play significant roles in cardiac homeostasis [21]. Interestingly, upregulation of Piezo1 expression levels was rapid and maximized at 15 min of exposure to 25 mM glucose (1.425 ± 0.05-fold relative to the control), decreasing thereafter (Figure 4a, upper panel, and Figure 4b).

Subsequently, we examined the time-dependent response of connexin 43 expression levels, with the latter being the primary protein involved in gap junction formation in the heart [33]. Thus, treatment of H9c2 cells with 25 mM glucose caused a relatively rapid and significant increase in connexin 43 expression levels, which maximized at 30 min (3.6 ± 0.1-fold relative to the control), remaining significantly elevated for 2 h (3.3 ± 0.1-fold relative to the control; ** *p* < 0.01 compared with the control values) (Figure 4a, second panel from top, and Figure 4c). Intriguingly, another band was also detected, most probably corresponding to GJA1-20k (gap junction protein alpha 1–20 kDa), the most abundant endogenous internally translated isoform of connexin 43. GJA1-20k also exhibited a considerable upregulation in its expression levels, with maximal values attained at 1 h (6.65 ± 0.33-fold relative to the control; ** *p* < 0.01 compared with the control values) (Figure 4a, third panel from top, and Figure 4d). Equal protein loading was verified by staining the membrane with Ponceau (Figure 4a, bottom panel).

### 3.5. p38-MAPK and ERK1/2 Are Rapidly Phosphorylated in High-Glucose-Treated H9c2 Cells

**Figure 5:**
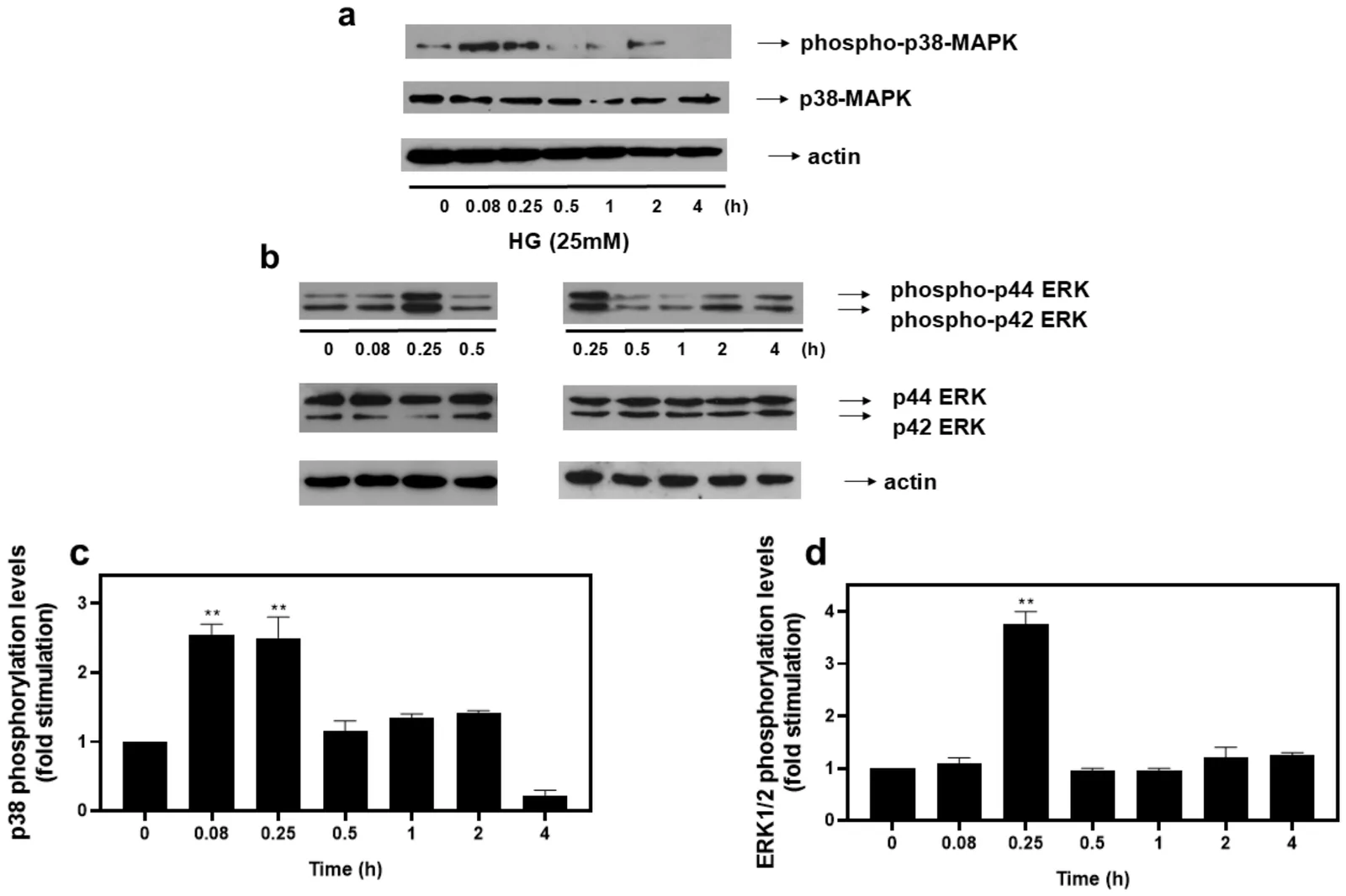
HG enhances p38-MAPK and ERK1/2 phosphorylation time-dependently. H9c2 cells were treated with 25 mM glucose (HG) for time intervals ranging from 5 min to 4 h, as indicated. Protein extracts (40 µg/lane) were subjected to SDS-PAGE and immunoblotted with (**a**) an anti-phospho-p38-MAPK antibody (**upper panel**) and (**b**) an anti-phospho-ERK1/2 antibody (**upper panels**). Respective samples were also incubated with antibodies against total p38 ((**a**) middle panel), total ERK1/2 ((**b**) middle panels) or actin ((**a**,**b**) bottom panels). Representative western blots of at least three independent experiments with overlapping results are shown. Quantification of protein bands was performed by scanning densitometry and plotted (respective graphs: (**c**) and (**d**)). Results are means ± SEM (*n* = 3). ** *p* < 0.01 compared with control values.

Exposure of cells to high glucose for at least 24 h or even longer has been previously reported [34] to cause activation of members of the MAPK superfamily of kinases. Therefore, it was of interest to explore their potential time-dependent phosphorylation pattern. To this end, performing western blot analysis, we detected an immediate and brief activation of p38-MAPK, which was maximized at 5–15 min (2.55 ± 0.16-fold relative to the control; ** *p* < 0.01 compared with control values) (Figure 5a, upper panel, and Figure 5c). We also observed a similarly direct activation of ERK1/2, evidenced by an augmentation in their phosphorylation levels at 15 min (3.75 ± 0.23-fold relative to the control; ** *p* < 0.01 compared with control values) (Figure 5b, top panels, and Figure 5d). No fluctuations in the kinases’ total expression levels were identified (Figure 5a,b, middle panels), with equal loading being confirmed by immunoblotting identical samples with a specific anti-actin antibody (Figure 5a,b, bottom panels).

### 3.6. Autophagy-Associated Signaling Is Promoted in High-Glucose-Treated H9c2 Cells

**Figure 6:**
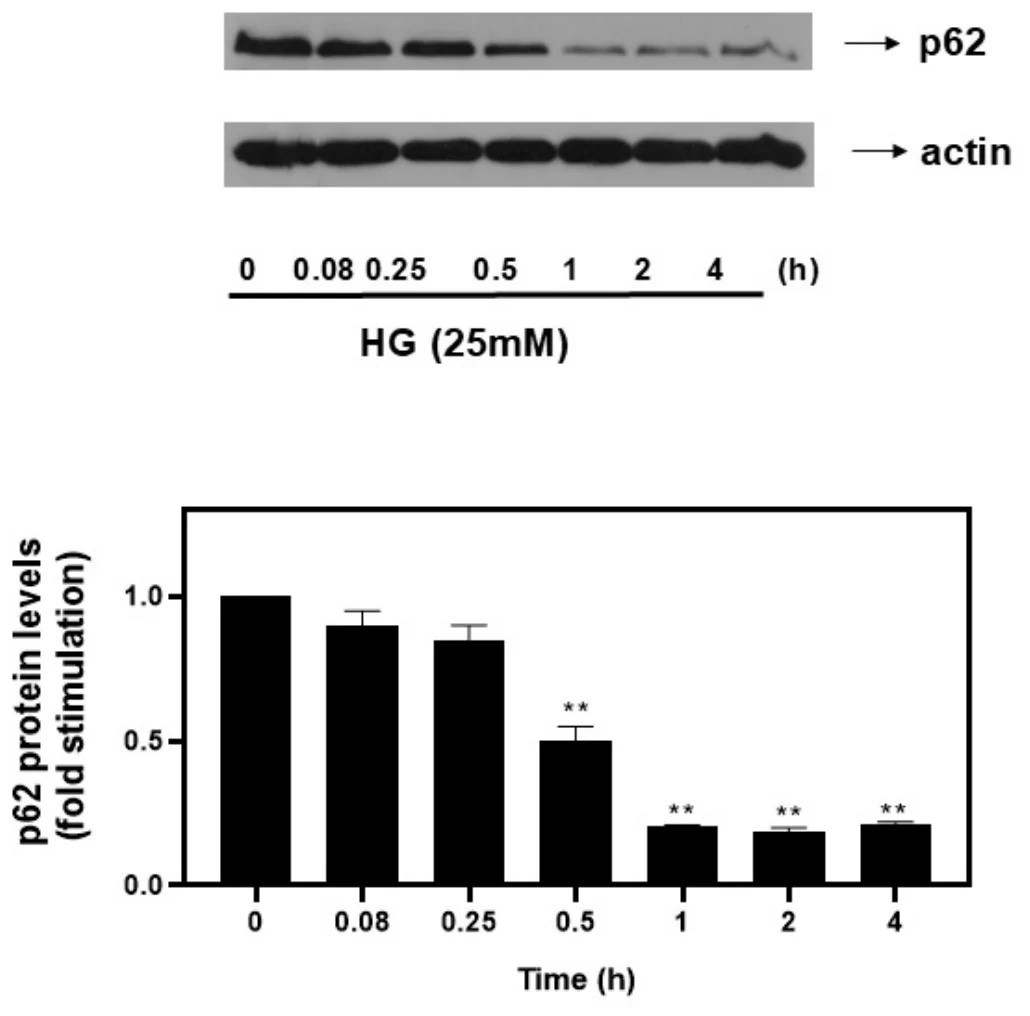
HG induces p62 degradation in a time-dependent manner. H9c2 cells were treated with 25 mM glucose (HG) for time intervals ranging from 5 min to 4 h, as indicated. Protein extracts (40 µg/lane) were subjected to SDS-PAGE and immunoblotted with an anti-p62 antibody (**upper panel**) and an anti-actin antibody (**bottom panel**). Part of the actin blot presented is also shown in Figure 3b (**left bottom panel**), since they correspond to identical sample lysates. The western blots shown are representative of at least three independent experiments with overlapping results. Quantification of protein bands was performed by scanning densitometry and plotted (respective graph). Results are means ± SEM for at least three independent experiments. ** *p* < 0.01 compared with control values.

Results regarding promotion or suppression of autophagy under hyperglycemic conditions can be contradictory depending on cell type, with most studies almost exclusively evaluating responses after at least 24 h of treatment. Therefore, we next explored the possibility of early onset of the autophagic mechanism in H9c2 cells treated with 25 mM glucose for time intervals ranging from 5 min to 4 h. Hence, expression levels of p62 were initially detected, since p62 degradation constitutes a hallmark of autophagy [35]. Of note, p62 levels were found to be significantly reduced after 30 min of treating H9c2 cells with 25 mM glucose (~by 50 ± 5.5% relative to the control), with their proteolysis being almost complete (~80 ± 2.3% relative to control) by 1 h (Figure 6, upper panel and graph). Equal loading was confirmed via immunoblotting with a specific anti-actin antibody (Figure 6, bottom panel).

**Figure 7:**
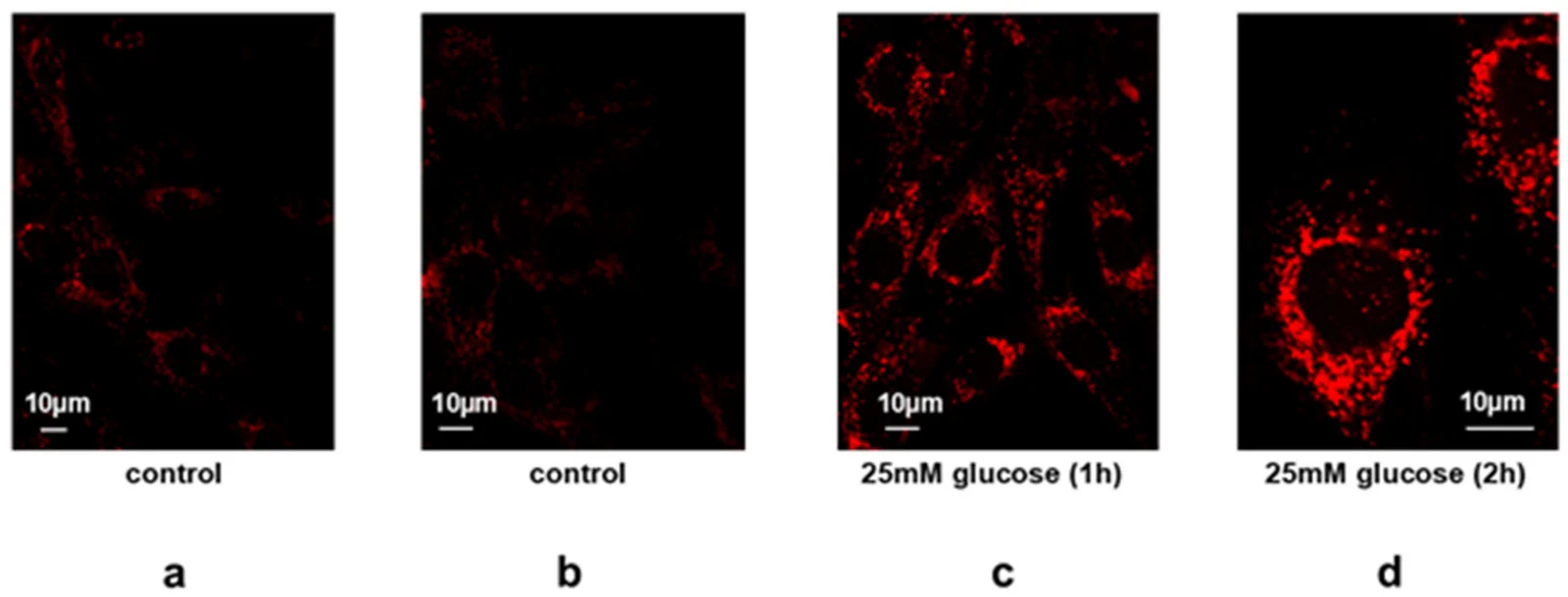
HG augments acidic autophagy-associated vesicles in H9c2 cells, evidenced by staining with acridine orange in untreated H9c2 cells ((**a**,**b**): control) as well as in cells treated with 25 mM glucose for 1 h (**c**) or 2 h (**d**). Staining was significantly enhanced, indicating induction of autophagy. Cells were visualized under a Zeiss Axioplan fluorescence microscope (bar scale: 10 μm).

Staining with acridine orange complemented the aforementioned results, with the number of orange–red acidic vesicles detected (most probably representing autolysosomes) progressively increasing after exposing H9c2 cells to 25 mM glucose for 1 or 2 h, compared with untreated (control) cells (Figure 7c and Figure 7d vs. Figure 7a and Figure 7b respectively).

**Figure 8:**
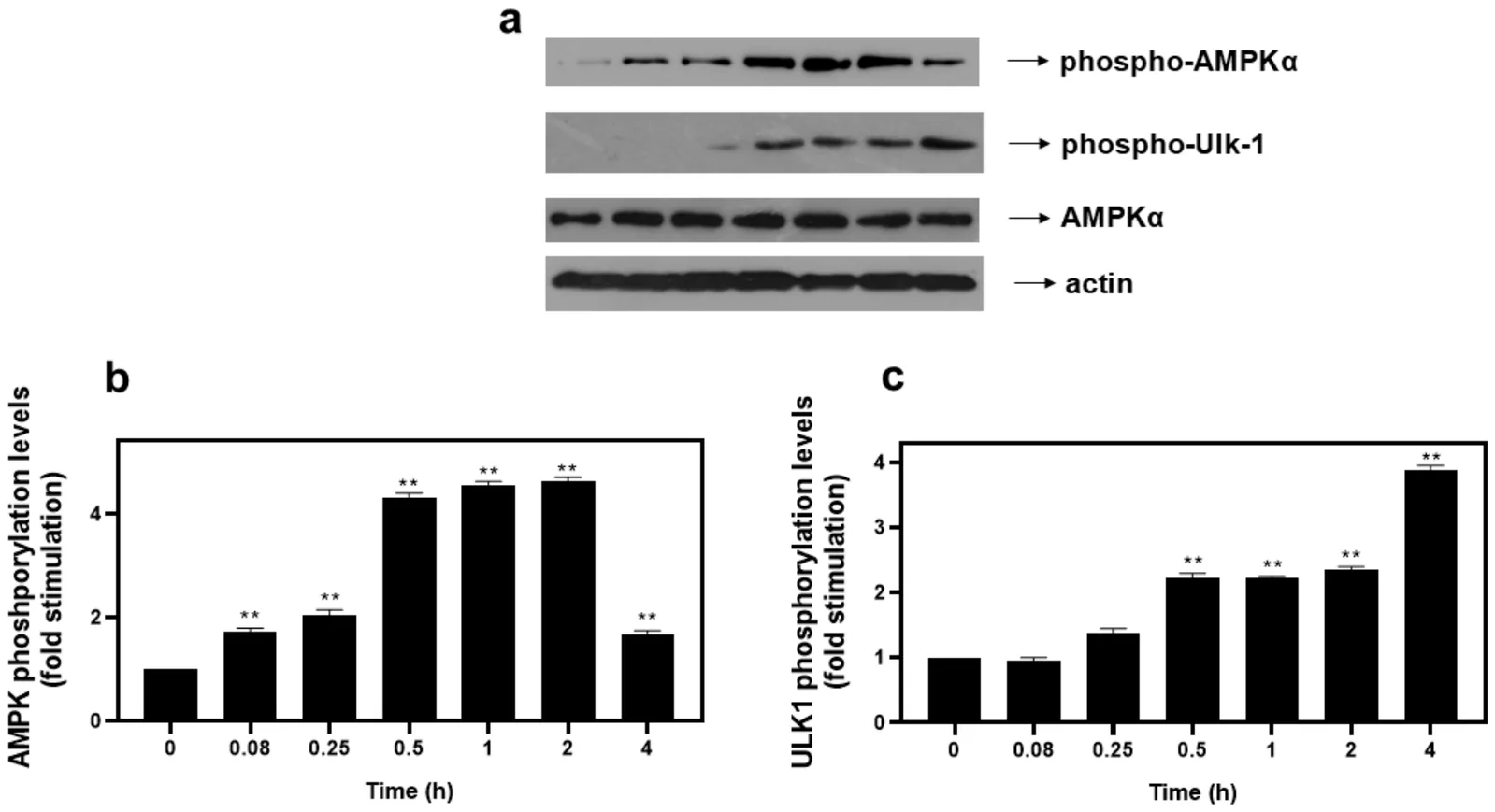
HG induces AMPK and ULK-1 phosphorylation in H9c2 cells. Cells were exposed to 25 mM glucose (HG) for time intervals ranging from 5 min to 4 h, as indicated. (**a**) Protein extracts (40 µg/lane) were subjected to SDS-PAGE and immunoblotted with an anti-phospho-AMPK antibody (**upper panel**) and an anti-phospho-ULK-1 antibody (2nd panel from top). Respective samples were next incubated with antibodies against total AMPK levels (3rd panel from top) or actin (**bottom panel**). Representative western blots of at least three independent experiments with overlapping results are shown. Quantification of protein bands was performed by scanning densitometry and plotted ((**b**) and (**c**), respectively). Results are means ± SEM (n = 3). ** *p* < 0.01 compared with control values.

Triggering of autophagy was also corroborated by the gradual upregulation in the phosphorylation levels of the alpha subunit of AMPK, as well as its downstream substrate Ulk-1, established mediators of the autophagic mechanism [36]. In particular, we observed an early upregulation in the phosphorylation levels of AMPK at 5 min (1.73 ± 0.13-fold relative to the control; ** *p* < 0.01 compared with control values), with maximal levels at 1–2 h (4.58 ± 0.15-fold relative to the control; ** *p* < 0.01 compared with control values), declining thereafter (Figure 8a, top panel, and Figure 8b). No fluctuations were detected in total AMPK levels (Figure 8a, third panel from top), with equal protein loading once again confirmed via immunoblotting analysis of actin levels (Figure 8a, bottom panel).

Regarding phosphorylation of Ulk-1, known as the mammalian autophagy-initiating kinase, this was found to be induced after 30 min (2.22 ± 0.1-fold relative to the control; ** *p* < 0.01 compared with control values), becoming maximal at 4 h (3.87 ± 0.12-fold relative to the control; ** *p* < 0.01 compared with control values) (Figure 8a, second panel from top, and Figure 8c).

### 3.7. High Glucose Enhances Bip Expression and eIF2 Alpha Phosphorylation in H9c2 Cells

**Figure 9:**
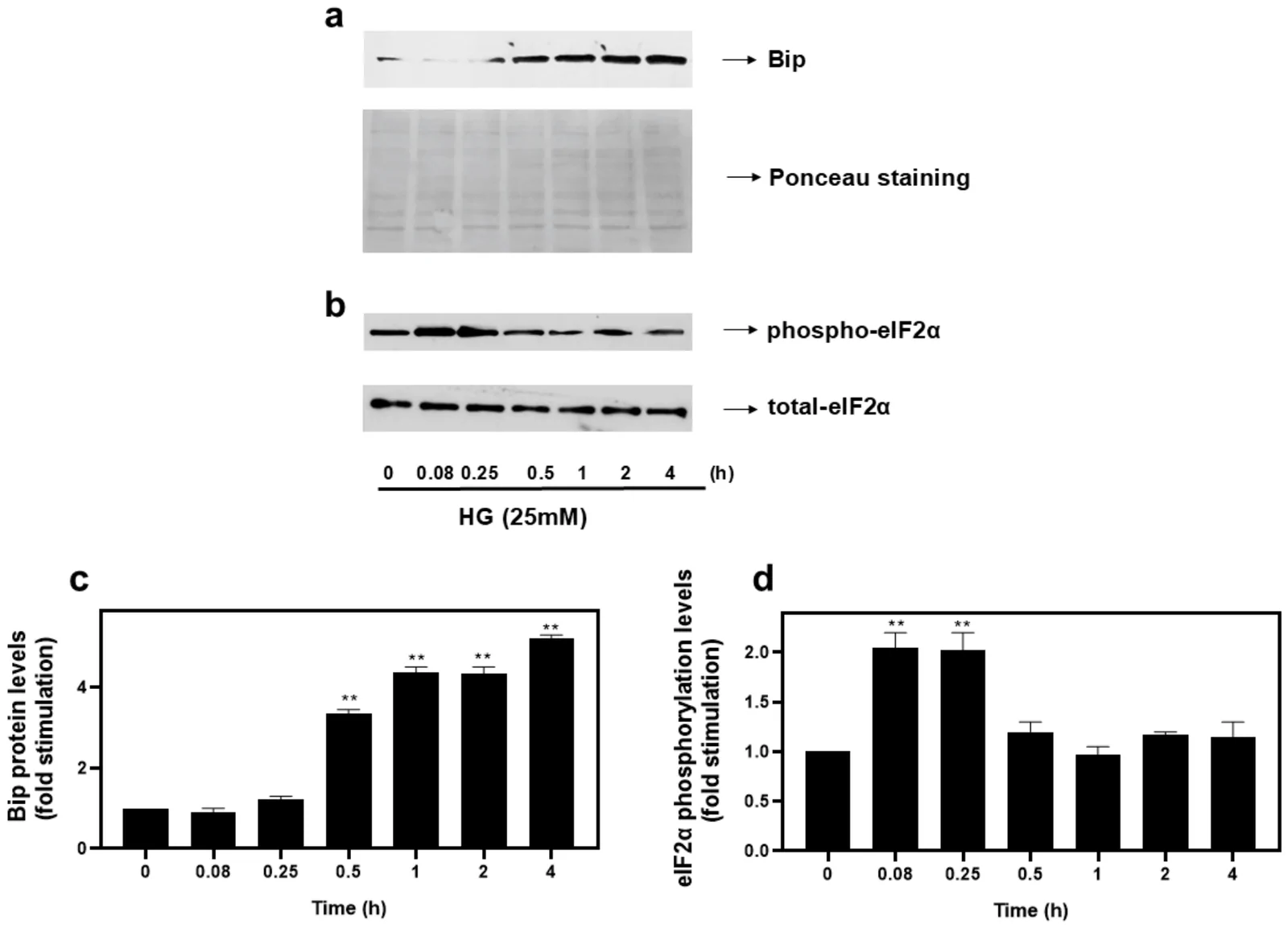
Bip expression and eIF2alpha phosphorylation are upregulated in H9c2 cells treated with 25 mM glucose for increasing time intervals. Cells were exposed to HG, and protein extracts (40 µg/lane) were subjected to SDS-PAGE and then immunoblotted with an anti-Bip antibody ((**a**) upper panel). Equal protein loading and transfer efficiency were verified via Ponceau staining of the respective membrane ((**a**) bottom panel). (**b**) Respective membranes were also incubated with antibodies against phosphorylated (**upper panel**) or total eIF2α levels (**bottom panel**). Representative western blots of at least three independent experiments with overlapping results are shown. Quantification of protein bands was performed by scanning densitometry and plotted ((**c**) and (**d**), respectively). Results are means ± SEM (*n* = 3). ** *p* < 0.01 compared with control values.

Given the established correlation in our laboratory of ER stress occurrence with IRS induction in H9c2 cells under conditions of hyperosmotic stress [37], but also after treatment with tunicamycin (0.1 μg/mL), an established ER stress chemical inducer (Supplementary Figure S1), we subsequently probed into the expression levels of Bip, an ER chaperone [38], and phosphorylation of the eIF2 alpha subunit, an IRS marker [18]. Bip expression levels were profoundly elevated after 30 min of treating H9c2 cells with 25 mM glucose (3.34 ± 0.17-fold relative to the control; ** *p* < 0.01 compared with control values), with maximal values attained after 4 h (5.22 ± 0.12-fold relative to control; ** *p* < 0.01 compared with control values) (Figure 9a, upper panel, and Figure 9c). Ponceau staining verified equal protein loading (Figure 9a, bottom panel). Of note, phosphorylation of the alpha subunit of eIF2 exhibited a rapid, transient and maximal increase at 5–15 min (2.25 ± 0.2-fold relative to the control; ** *p* < 0.01 compared with control values) (Figure 9b, top panel, and Figure 9d), with no fluctuations in total levels of eIF2α (Figure 9b, bottom panel).

### 3.8. High-Glucose-Induced Connexin 43 and GJA1-20k Upregulation Is ERK1/2-Dependent and ER-Stress-Mediated

With emerging studies highlighting fluctuations in the expression pattern of connexins under persistent hyperglycemic conditions [39,40], identifying potential effectors that may mediate this modulation is of great significance. In this context, taking into consideration the observed activation time profiles of ERK1/2, p38-MAPK and ER-stress-associated signaling, we made an effort to probe their potential effects on connexin 43 and GJA1-20k upregulation in H9c2 cells exposed to 25 mM glucose.

**Figure 10:**
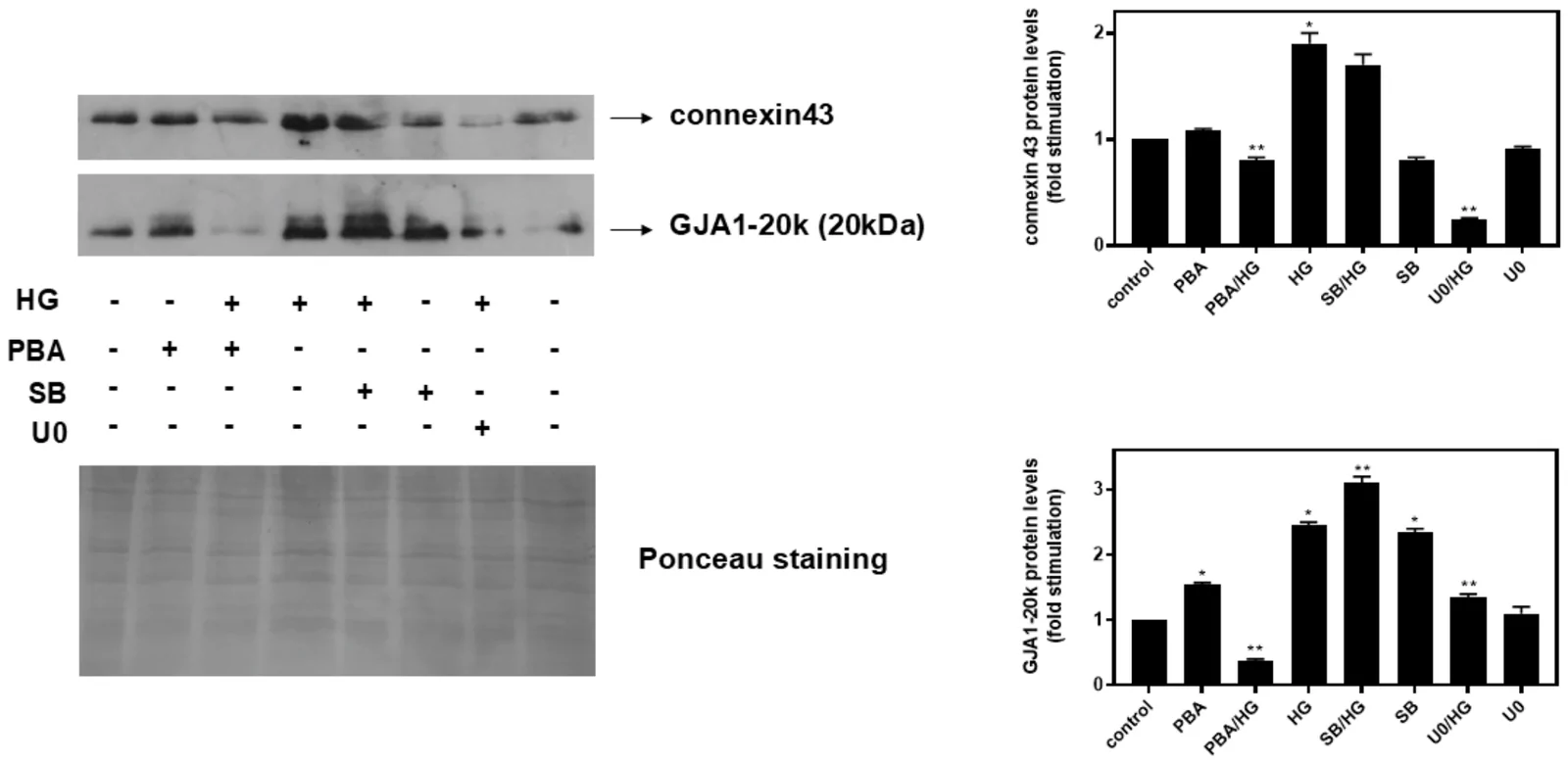
PBA and U0126 attenuate HG-induced connexin upregulation in H9c2 cardiac cells. H9c2 cells were left untreated (control), treated with 25 mM glucose (HG), or pre-incubated with PBA, SB203580, or U0126 for 30 min and then exposed to 25 mM glucose in the presence of the inhibitors (PBA, SB, and U0), or were exposed to the inhibitors alone. Cell extracts (40 µg/lane) were subjected to SDS-PAGE and immunoblotted with an antibody against connexin 43 (**upper panel**), also detecting GJA1-20k (**middle panel**). Ponceau staining of the membrane was also performed (**bottom panel**). Representative western blots of at least three independent experiments with overlapping results are shown. Quantification of protein bands was performed by scanning densitometry and plotted (respective graphs). Results are means ± SEM (*n* = 3). * *p* < 0.01 compared with control values; ** *p* < 0.01 compared with HG-treated cells in the absence of the inhibitors.

Interestingly, as shown in Figure 10, while induction of connexin 43 by high glucose was not affected in the presence of SB203580 (a p38-MAPK inhibitor—10 μM) (Figure 10, upper panel, fifth lane), it was considerably repressed in the presence of PBA (ER stress inhibitor—5 mM) (Figure 10, upper panel, third lane) and was totally overturned in the presence of U0126 (an inhibitor of the MEK1/2-ERK1/2 pathway—10 μM) (Figure 10, upper panel, seventh lane, and respective graph). Looking into the respective effects of these compounds on GJA1-20k expression, they were found to be similar, since PBA was also detected to suppress GJA1-20k high-glucose-induced expression (Figure 10, middle panel, third lane, and respective graph), as was also the case for U0126 (Figure 10, middle panel, seventh lane), while SB203580 rather enhanced it (Figure 10, middle panel, fifth lane). Connexins subcellular distribution was then determined via immunofluorescence confocal microscopy.

**Figure 11:**
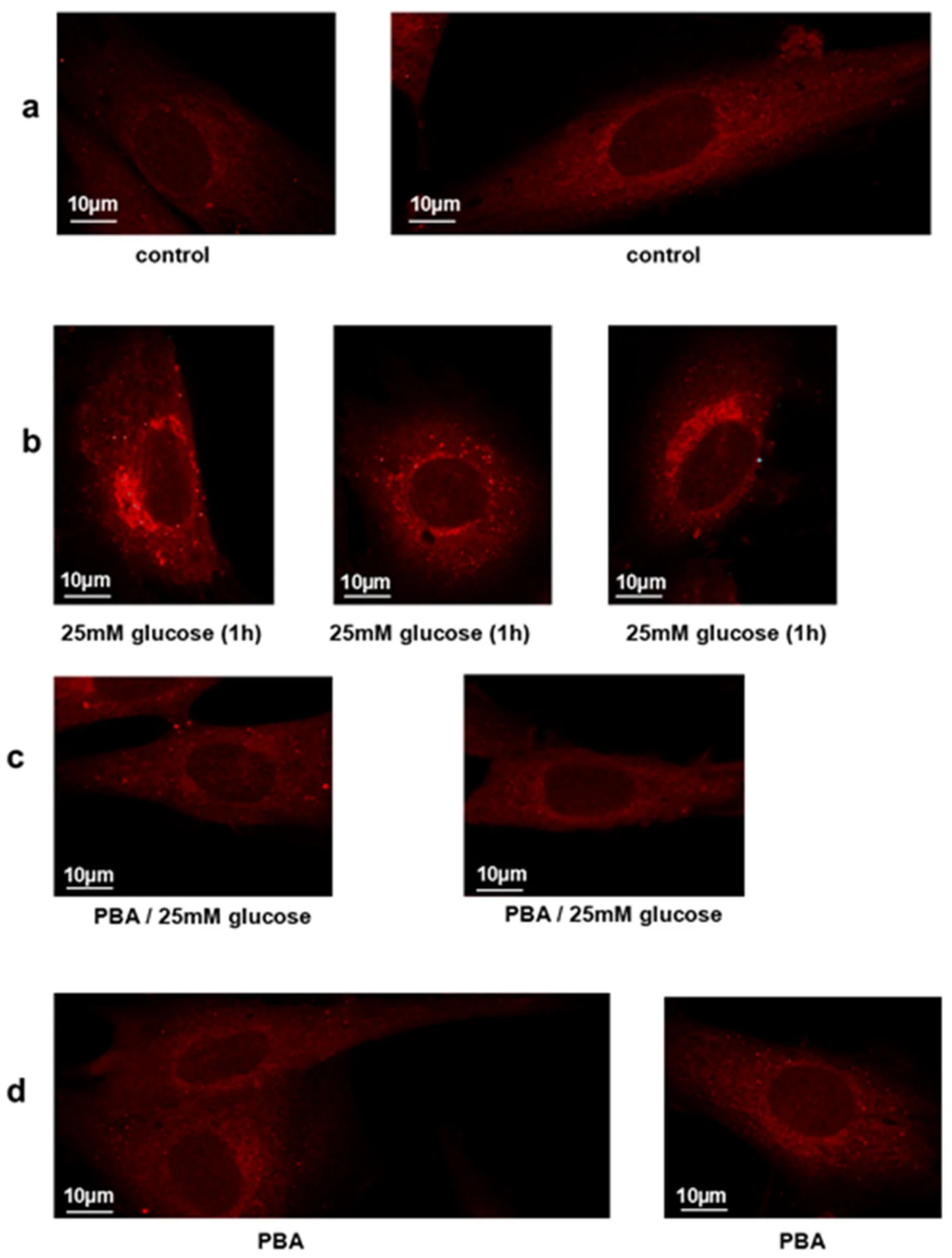
Immunofluorescence assessment of connexins localization pattern revealed that high glucose enhanced expression levels and perinuclear accumulation of connexins in an ER-stress-associated manner. H9c2 cells were left untreated (control-(**a**)), incubated with 25 mM glucose (**b**) or PBA alone (**d**), or pre-treated with PBA and then exposed to high glucose (PBA/high glucose (**c**)). After fixation and permeabilization, cells were incubated with an anti-connexin 43 antibody, followed by incubation with a TRIC-conjugated secondary antibody (red). Cells were then visualized under a Zeiss Axioplan fluorescence microscope (bar scale: 10 μm). Images are indicative of at least three independent experiments with similar results.

Interestingly, in agreement with the western blotting results, connexin immunoreactivity was potently enhanced in H9c2 cells exposed to high glucose (Figure 11b) compared with untreated cells (Figure 11a) or cells treated with PBA alone (Figure 11d) or cells pre-treated with PBA and subsequently exposed to high glucose in the presence of the inhibitor (Figure 11c). Of note, high glucose appeared to induce a perinuclear accumulation of connexins immunofluorescence, which was distributed across the cytoplasm in all other conditions examined (Figure 11).

At this point, it should be noted that all the aforementioned findings were confirmed to be primarily attributed to glucose-related effects rather than to the osmotic input conferred by HG, since mannitol at 25 mM either did not stimulate the mechanisms reported herein at all, or did so in a time-dependent differential manner (indicative data shown in Supplementary Figures S2–S8).

## 4. Discussion

An association between hyperglycemia and cardiac pathogenicity has long been acknowledged [41]. Cardiovascular disorders are recognized as a major contributor to the mortality and clinical complications exhibited in patients with diabetes, independently of the particular traits that accompany particular types of the metabolic syndrome. Intriguingly, studies probing into the mechanisms mediating the detrimental effects of hyperglycemia almost exclusively revealed the long-term responses initiated under these conditions, rather than the early onset of signaling events culminating in the dysfunctions ultimately observed. Hence, the present study aimed to decipher the early responses activated by high glucose in H9c2 cardiac cells, along with their potential interrelations, molding, in a decisive manner, the ultimate outcome.

In agreement with the established notion that hyperglycemia exerts a deleterious effect on the myocardium, H9c2 cardiac cells treated with increasing doses of glucose exhibited an equally increasing repression of viability (Figure 1a). In their studies, Pandey et al. (2022) [42] and Varela et al. (2021) [43] also reported the direct correlation of elevated glucose concentrations with H9c2 cellular death mainly via ROS upregulation. Accordingly, in the present study, retention of apoptosis and oxidative stress conditions by ZVAD-FMK and melatonin, respectively, partly restored cell viability. It was of great interest that we also detected the harmful impact of PBA, bafilomycin and U0126 on H9c2 viability during exposure to high glucose, underlying the potential beneficial role of ERK1/2 activation and ER-stress- and autophagy-related signaling under hyperglycemic conditions (Figure 1b). Conflicting reports have marked ER stress as either exerting a salutary effect via suppression of translation, when the ER is overloaded, or playing a detrimental role, in the case that the translational halt impacts essential survival effectors (reviewed in [44]). With reference to autophagy, contradicting our data, Kobayashi et al. (2012) observed that high glucose (17 or 30 mM) suppressed autophagy in rat neonatal cardiomyocytes or adult mice, promoting an adaptive beneficial response against the HG pro-death effect [6]. However, one needs to point out that this salutary restraint of the autophagic mechanism was observed after 72 h of exposure to the toxic doses of glucose used, while our data underlined autophagy’s beneficial impact in the context of cellular responses triggered within 24 h of treatment with HG. Additionally, in accordance with our findings regarding MAPK’s involvement in high glucose’s impact on cardiac cell viability, ERK1/2 have been shown to be activated in a short-term hyperglycemic experimental model (4 weeks after streptozotocin treatment), while being inhibited under long-term hyperglycemia (20 weeks after streptozotocin treatment), eliciting heart protection vs. dysfunction, respectively [45,46].

Subsequently, an effort was made to further explore and elucidate the signaling pathways mediating these mechanisms. In this respect, the effect of high glucose (HG) on established effectors of apoptosis was initially investigated. Interestingly, maximal proteolysis of PARP, as well as caspase-3, was attained at 2 h (Figure 2), with occurrence of apoptosis detected up to that date, primarily as a long-lasting effect [10,42,47].

Given the plethora of reports noting enhancement of ROS generation by HG [10,48,49], we next set out to detect the expression time profile of potential effectors involved. With only a few existing studies focusing on the role of cytochrome P450 oxidoreductase (POR) in oxidative stress generation, we initially assessed its time-dependent profile, which was found to be enhanced, persistent and maximal at 4 h (Figure 3a,c). Notably, heme oxygenase-1 (HOX-1) exhibited a similar time-dependent profile (Figure 3b,d). With POR having an established role mainly as a driver of ferroptosis and HOX-1 validated as a primary effector of antioxidative defensive mechanisms, any interconnection between them is sparsely acknowledged. Hence, *to our knowledge, the present study is the first time their potential interrelation has been revealed in cardiac cells exposed to high glucose.* This could be attributed to POR serving as a mandatory electron donor to HOX-1 so that the latter can operate [50]. Zhao et al. (2013) have also reported HOX-1 induction in streptozotocin-diabetic mice, while, to the best of our knowledge, *there is no record of the direct effect of HG on POR expression levels to date* [51].

Taking into consideration the fact that high glucose levels function not only as a metabolic and oxidative stress stimulus but also as a biomechanical stressor, due to the hyperosmotic impact they confer, we next made an effort to assess HG’s effect on the expression of Piezo1 and connexin 43, known for their involvement in mechanotransduction [21,52]. Piezo1 showed a strikingly rapid upregulation in its expression as early as 5 min after beginning H9c2 treatment with HG (Figure 4a, upper panel, and Figure 4b). Piezo1 has been previously shown to be increased in samples from human and mouse T2DM models [53]. In agreement, Niu et al. (2025) also reported the enhanced expression of Piezo1 in the hearts of T1DM and T2DM diabetic mice, as well as in high-glucose-treated H9c2 cells [54].

In the present study, expression of connexin 43 was also observed to increase, attaining maximal values at 30 min, remaining elevated for at least 2 h (Figure 4a, second panel from top, and Figure 4c). Accordingly, connexin 43 was increased in mouse retinal microvascular endothelial cells, in the retinas of diabetic mice [55] and in H9c2 cells treated with 25 mM glucose for 24 h [3]. Conversely, many studies have highlighted suppression of connexin 43 in rat microvascular endothelial (RME) cells (30 mM glucose for 9 days) [56], in glomerular mesangial cells [57], and in human cortical bone samples from T2DM patients and MLO-Y4 cells [58]. Since connexin 43 expression is repressed in human failing hearts [59], its decrease is concomitant with myocardial dysfunction, while its increase presumably indicates an attempt for preservation of homeostasis. *With the time-dependent pattern of connexin 43 expression not being previously assessed in the initial stages of hyperglycemia, further studies are required to fully elucidate the cell-type and dose-dependent respective responses induced*. The mechanisms regulating connexin 43 expression are quite complex and are executed not only at the translational and post-translational levels (by phosphorylation, ubiquitination, and SYMOylation) but also via phosphorylation and modifications regarding its final delivery to gap junctions via a respective trafficking machinery [60]. Any perturbations or disturbances may disrupt delivery, hence the function of connexin 43.

Unexpectedly, using the anti-connexin 43 antibody, another protein band was also detected at ~20 kDa, with an equally positively responsive expression profile under conditions of HG. The antibody used in the current study was raised against a peptide corresponding to “a C-terminal segment of the cytoplasmic domain of human and rat connexin 43”. Of the six truncated isoforms of Cx43 that have been identified, GJA1-20k, a 20 kDa naturally occurring fragment of connexin 43, is the most abundantly expressed in the human heart and multiple cell lines (MEF, A549, and HEK293) and corresponds to the entire carboxyl-terminal (CT) domain of connexin 43, plus the ending part of its fourth transmembrane domain [61,62,63,64,65]. Therefore, it is highly likely that the band detected in our experiments corresponds to GJA1-20k. GJA1-20k was found to be induced by hypoxia in the mouse brain [66] and after ischemia, ischemia/reperfusion or inhibition of the mTOR pathway in the heart, suggesting its potential cardioprotective role [62,63,64]. *Most importantly, GJA1-20k*, also referred to as “the tail” of connexin 43, *has not been previously reported to be upregulated by HG in cardiac cells, a finding that could signify enhancement of its function under HG conditions, i.e., acting as a stress-responsive protein*, delivering the similarly upregulated connexin 43 to the desired end-points of its trajectory and preserving mitochondrial homeostasis, cytoskeletal organization, etc.

In accordance with our MTT data and the notion that MAPKs play a nodal role in diabetes-induced heart pathogenicity [46,67], the rapid and relatively transient phosphorylation, and thus activation, of p38-MAPK (Figure 5a,c), as well as of ERK1/2 (Figure 5b,d), was detected in the present study. Interestingly, reports have underlined ERK1/2 activation in the context of diabetic cardiomyopathy, mainly attributed to ROS generation under these conditions [68]. Nevertheless, discrepancies exist regarding the salutary or detrimental role ERK1/2 may play [46]. Activation of ERKs conferring protection has been established in cardiac hypertrophy, as well as in the context of the hyperglycemic myocardium [69,70]. Meanwhile, in line with our data, Igarashi et al. (1999) detected p38-MAPK activation in rat aortic smooth-muscle cells exposed to mild or severe hyperglycemia [71], with Nakagami et al. (2001) also reporting p38-MAPK activation in HG-treated human aortic endothelial cells [72]. P38-MAPK may also play a beneficial or detrimental role, if any, depending on the experimental setting under investigation.

As far as autophagy is concerned, we probed into the time-dependent expression profiles of a number of autophagic markers and demonstrated induction of autophagy (Figure 6, Figure 7 and Figure 8). In contrast, when p62 degradation in conjunction with AMPK/ULK-1 axis activation was assessed, it was found to be suppressed in rat neonatal cardiac myocytes exposed to high glucose for 72 h [6]. In addition, Xie et al. (2011) also demonstrated autophagy inhibition in diabetic OVE26 mice [73]. Nevertheless, Kanamori et al. (2015) have verified that autophagy is enhanced in type 1 diabetic mouse hearts but suppressed in type 2 diabetic mouse hearts [74]. Considering the notion that “stimulation of autophagy may serve as a novel therapeutic protocol against diabetic cardiomyopathy”, in conjunction with the early onset of autophagy determined in the present study, further investigation of the underlying mechanisms appears to be of the utmost importance.

Autophagy-related signaling pathways can function in parallel or independently of ER-stress-associated mechanisms, with their interplay established in a number of pathophysiological disorders [75] but not yet fully elucidated. In accordance with studies detecting phosphorylation of eIF2 alpha by high glucose in a human leukemic monocytic cell line (THP-1) and diabetic human subjects [76], our study revealed an immediate and transient phosphorylation of the eIF2 alpha subunit, along with time-dependent upregulation of Bip expression (Figure 9). Interestingly, when treating H9c2 cells with tunicamycin, an established ER stress chemical inducer, even at a relatively low dosage (0.1 μg/mL), we also detected an immediate augmentation in eIF2a phosphorylation and a significant upregulation in Bip expression levels (data illustrated in Supplementary Figure S1). Hence, in this particular experimental model, these two effectors appear to be responsive and closely linked to ER stress. Further studies are required, however, to unveil any actual interconnection between high-glucose-induced ER stress and autophagy-related signaling in our experimental setting. Intriguingly, although induction of autophagy upon ER stress has been demonstrated, existing reports have yet to decipher and identify the definite nature of the relationship between the two [77].

With connexins found to be markedly upregulated under HG conditions in H9c2 cells in the present study, we next sought to identify potential mechanisms and effectors involved. Since MAPKs have been reported to contribute to connexins regulation [78,79], and given their activation in the present study, it was of interest to look into their potential participation in connexin 43 and GJA1-20k induction. Astonishingly, as illustrated in Figure 10, ERKs inhibition obliterated HG-induced connexin 43 increase and halved GJA1-20k upregulation. Meanwhile, p38-MAPK repression by SB203580 had no statistically significant effect. Interestingly, reports have underlined ERKs-mediated connexin 43 regulation, mainly via phosphorylation at multiple serine residues [80]. In addition, in conjunction with other kinases such as Src and Akt, ERK1/2 coordinate connexin 43-related turnover at, or disassembly of, gap junctions [79]. Nevertheless, ERK1/2-mediated *suppression of connexin 43 expression has not been previously demonstrated in HG-treated cardiac cells.* This was also the case for GJA1-20k. The signaling pathways ultimately affecting GJA1-20k regulation constitute a hot topic of ongoing research. In particular, in neonatal mouse cardiomyocytes, repression of the Akt/mTOR axis resulted in increased expression of GJA1-20k [81], with Mnk1/2 (MAP kinase-interacting serin/threonine-protein kinase ½) inhibition found to augment endogenous GJA1-20k expression in primary and cancer cells [61]. In agreement with our findings, an ERK1/2-dependent decrease in GJA1 20k expression has been reported in mouse mammary epithelial cells [82]. Additionally, in aging mouse hearts, enhanced activation of p38-MAPK correlated with a decrease in GJA1-20k expression [83], matching increased expression of GJA1-20k in HG-treated H9c2 cells in the presence of SB203580 (Figure 10).

Interestingly, the *ER-stress-related contribution to connexins regulation was also verified for the first time in cardiac cells exposed to HG in the present study.* Hence, PBA drastically constrained HG-induced expression levels of both connexin 43 and GJA1-20k (Figure 10). Of note, Bai et al. (2025) have noted that ER stress induces connexin 26 ubiquitination and subsequent progressive degradation in the cochlea of aged mice [84]. Nevertheless, one should also underline the reciprocal nature of this relation, since overexpression of connexin 43 may intracellularly transduce the ER stress insult from one hepatocyte to an adjacent one, exacerbating ER stress complications, and thus intensifying hepatosteatosis and insulin resistance [85]. Given the cytoprotective role of connexins under stress conditions, supplementary studies are essential to delineate and clarify the underlying mechanisms boosting connexins expression to preserve cell homeostasis.

Considering that changes in the expression levels of connexins often indicate modulation in their subcellular distribution, the latter was subsequently assessed. Hence, although the anti-connexin 43 antibody does not discriminate between connexin 43 and GJA1-20k, connexin’s immunofluorescence distribution pattern revealed a uniform localization throughout the cytoplasm under control conditions, with high glucose observed to enhance dispersion of connexins mainly in the perinuclear compartment (Figure 11). This finding correlates with a study by Barreto et al. (2024) reporting connexin 43’s perinuclear distribution in H9c2- and iPSC-derived cardiomyocytes exposed to an inflammatory microenvironment [86]. Martins-Marques et al. (2023) also revealed the non-canonical distribution of connexin 43 away from gap junctions, indicative of its “non-canonical biological roles” in HEK293 and adult primary cardiomyocytes [87]. In particular, they demonstrated connexin 43 to form channels at the nuclear envelope, possibly mediating nucleocytoplasmic shuttling of small molecules. Our findings on connexins’ subcellular distribution under hyperglycemic conditions highlight the potential role these molecules may play in ER-to-nucleus transport, likely impacting transcriptome modulation or signal transduction.

**Figure 12:**
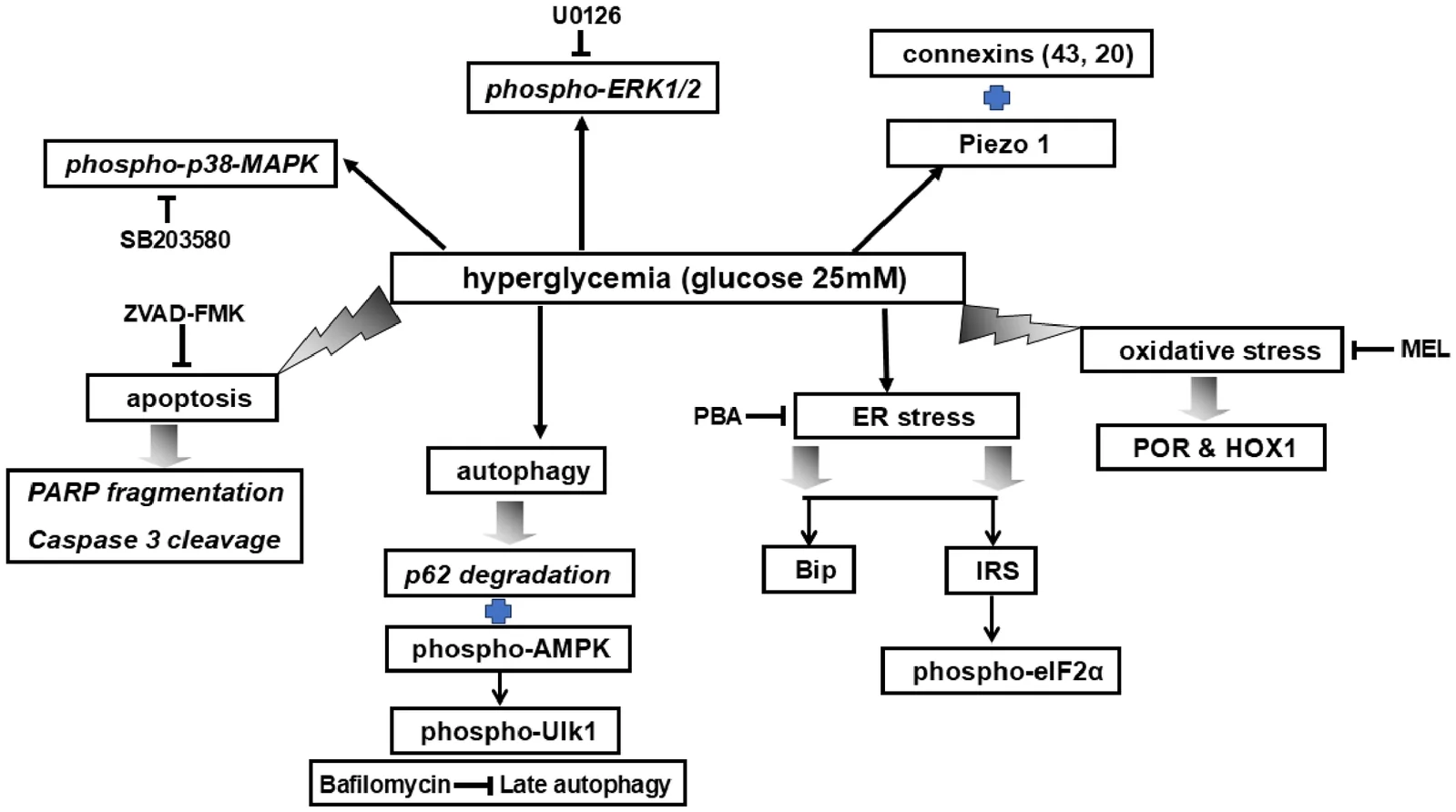
Schematic diagram illustrating signaling pathways and mechanisms activated by 25 mM glucose in H9c2 cells. 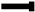 inhibition; 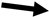 activation of detrimental pathway.

Collectively, the present study elucidated the early phase of cardiac cells’ responses to HG, revealing adaptive signaling pathways, underestimated as of yet, along with their potential interconnections (depicted in Figure 12).

**Figure 13:**
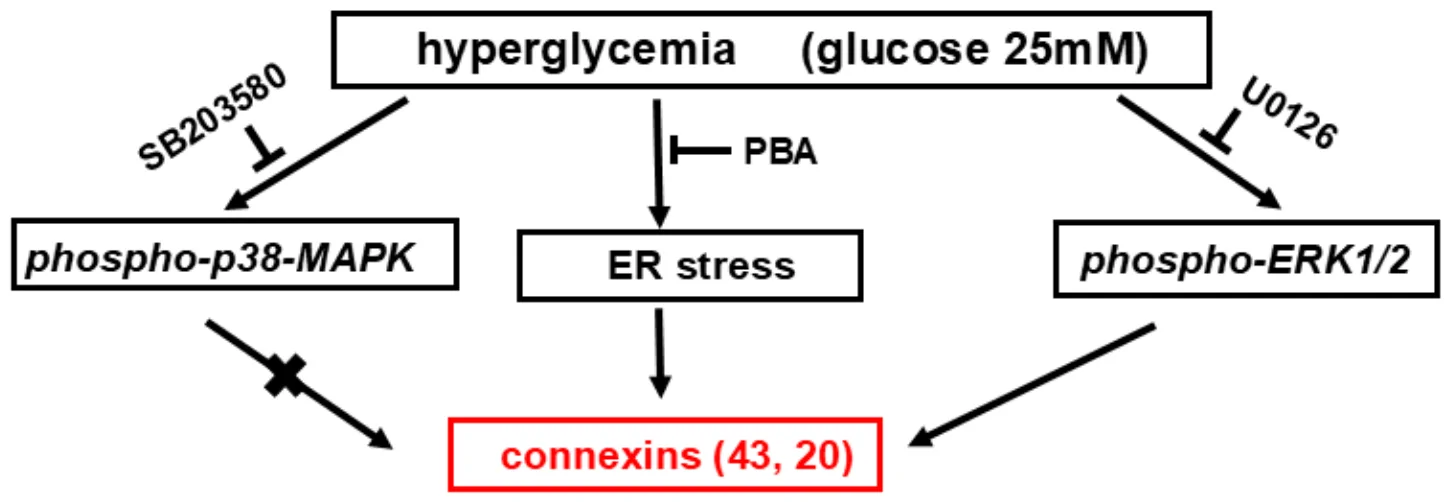
Schematic diagram depicting the different routes via which the HG signal ultimately impacts connexins. 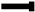 inhibition; 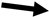 activation; and 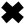 interruption of signal transduction.

The particular pathways that were found to affect connexins expression levels in HG-treated H9c2 cells are also illustrated in Figure 13.

With the clinical complications of hyperglycemia affecting an increasing number of patients, identification of the immediately initiated signaling cascades may reveal alternative routes either mitigating or propagating the detrimental insult. Characterization of effectors coming into play may contribute to development of interventions that could safeguard and ensure myocardial survival under hyperglycemic deleterious conditions. It should, nevertheless, be noted that any direct extrapolation of our findings to the complex and multifactorial context of hyperglycemic signal transduction footprint and its accompanying detrimental impacts on cardiac physiology would be speculative if not precarious. Cell-based in vitro studies cannot simulate the intricate and multifaceted microenvironment of the myocardium in a definite and unquestionable manner. Further experimentation is, therefore, essential to advance our understanding of the pivotal molecular effectors participating in the early cardiac responses triggered under hyperglycemic conditions, as well as their potential interconnections. In addition, although our findings revealed that mannitol, as an osmotic control, exerted completely different effects, ranging from zero to minimal induction of the signal transduction effects conferred by high glucose (data shown in Supplementary Figures S2–S8), the potential input of hyperosmolarity to the responses observed cannot be ruled out completely. Hence, additional studies are required in order to unveil definite glucose-specific effects. Nonetheless, characterization of the signal transduction routes promptly activated under hyperglycemic conditions in H9c2 cells in the present study may still provide insight into potential novel therapeutic targets, revealing, for the first time, the yet underestimated contribution of biomechanical sensors to the responses initiated.

## 5. Conclusions

Overall, to the best of our knowledge, *the present study outlines, for the first time, the signaling mechanisms promptly triggered by hyperglycemia in H9c2 cardiac cells* within a time frame of 5 min to 4 h. *Disclosure of the potential involvement of connexin 43 and GJA1-20k in the responses observed* may yield promising insights regarding formulation of novel salutary interventions in the context of hyperglycemia-related myocardial pathologies.

## Data Availability

The data presented in this study are available upon request from the corresponding author.

## Supplementary Materials

The following supporting information can be downloaded at https://www.mdpi.com/article/10.3390/cells15181651/s1. Figure S1: Treatment of H9c2 cells with 0.1μg/ml tunicamycin induces Bip expression as well as eIF2alpha phosphorylation in a time-dependent manner. Figure S2: Time-dependent pattern of mannitol–induced PARP proteolysis and caspase 3 cleavage in H9c2 cardiac cells. Figure S3: Time-dependent profile of mannitol-induced POR and HOX-1 expression in H9c2 cardiac cells. Figure S4: Mannitol does not significantly impact connexins expression levels. Figure S5: Mannitol enhances ERK1/2 but not p38-MAPK phosphorylation time-dependently. Figure S6: In H9c2 cells treated with 25 mM mannitol p62 degradation is not induced, AMPK phosphorylation is mildly triggered, while Ulk-1 is not phosphorylated. Figure S7: Bip upregulation and eIF2alpha phosphorylation in H9c2 cells treated with 25 mM mannitol for increasing time intervals. Figure S8: PBA suppresses Bip upregulation and eIF2alpha phosphorylation induced by 25mM glucose in H9c2 cells.

## Abbreviations

The following abbreviations are used in this manuscript:

BSA	Bovine serum albumin
CVDs	Cardiovascular disorders
DMSO	Dimethyl sulfoxide
DTT	Ditheiothreitol
ECL	Enhanced chemiluminescence
GJA1-20k	Gap junction protein alpha 1–20 kDa
MAPK	Mitogen-activated protein kinase
PARP	Poly(ADP-ribose) polymerase
ROS	Reactive oxygen species
TBS	Tris-buffered saline
